# Supplementary material for: Above-room-temperature strong intrinsic ferromagnetism in 2D van der Waals Fe3GaTe2 with large perpendicular magnetic anisotropy
Source: Nat Commun. 2022 Aug 29;13:5067. doi: 10.1038/s41467-022-32605-5 (PMC9424191; doi:10.1038/s41467-022-32605-5)
Supplement: Supplementary file 1 — Supplementary Information [file 41467_2022_32605_MOESM1_ESM.pdf]

Supplementary Information for

Above-room-temperature strong intrinsic ferromagnetism in 2D van der Waals Fe_3GaTe_2 with large perpendicular magnetic anisotropy

Gaojie Zhang[1,2,†], Fei Guo[3,†], Hao Wu[1,2], Xiaokun Wen[1,2], Li Yang[1,2], Wen Jin[1,2], Wenfeng Zhang[1,2,4], Haixin Chang[1,2,3,4,*]

[1]Center for Joining and Electronic Packaging, State Key Laboratory of Material Processing and Die & Mold Technology, School of Materials Science and Engineering, Huazhong University of Science and Technology, Wuhan 430074, China.

[2]Institute for Quantum Science and Engineering, Huazhong University of Science and Technology, Wuhan 430074, China.

[3]Liuzhou Key Lab of New-Energy Vehicle Lithium Battery, School of Microelectronics and Materials Engineering, Guangxi University of Science and Technology, Liuzhou 545006, China.

[4]Shenzhen R&D Center of Huazhong University of Science and Technology (HUST), Shenzhen 518000, China.

†These authors have contributed equally to this work.

[*]Corresponding author. E-mail: hxchang@hust.edu.cn

This Supplementary Information file includes:

1. Supplementary Notes 1-3
2. Supplementary Figures 1-14
3. Supplementary Tables 1-7
4. Supplementary References 1-37

Content:

Supplementary Notes..3

Supplementary Figures 8

Supplementary Tables...22

Supplementary References...29

Supplementary Note 1. XPS analysis of the Fe_3GaTe_2 crystal.

Before the XPS measurement, an exfoliated fresh Fe_3GaTe_2 crystal were quickly put into the vacuum XPS chamber to avoid oxidation. Except for the C element (C 1s, 285 eV) used to calibrate the peak position, only Fe, Ga, and Te were detected from the spectra of the Fe_3GaTe_2 crystal.

As shown in Supplementary Fig. 3a, the high-resolution Fe 2*p* spectra was deconvoluted into Fe $2p_{3/2}$ and Fe $2p_{1/2}$. The two peaks at 706.9 and 720.0 eV originate from the metallic iron (Fe^0) in Fe_3GaTe_2. The two other doublets with binding energies of 710.8 and 724.4 eV (with 713.7 and 727.3 eV satellites) can be ascribed to Fe^{3+} in Fe_3GaTe_2. Further, the area ratio of Fe^{3+} peaks and Fe^0 peaks is calculated as 2.08:1, which very close to the theoretical ratio 2:1 and corresponded to the Fe-I (Fe^{3+}) and Fe-II (Fe^0) in Fe_3GaTe_2. Meanwhile, the analysis of the high-resolution Ga 2*p* spectra in Supplementary Fig. 3b shows Ga $2p_{3/2}$ and Ga $2p_{1/2}$ peaks with binding energies of 1117.6 and 1144.5 eV. Similarly, Te phase (Te^{2-}) in the high-resolution Te 3*d* spectra was also deconvoluted into Te $3d_{5/2}$ and Te $3d_{3/2}$ with binding energies of 572.8 and 583.2 eV, respectively (Supplementary Fig. 3c).

Supplementary Note 2. Room-temperature magnetic anisotropy of the Fe_3GaTe_2 crystals.

The magnetic anisotropy of Fe_3GaTe_2 bulk crystals is analyzed by magnetic field dependent magnetization (M-H) tests at 300 K. As shown in Fig. 2b-d (main text) and high in-plane magnetic field test (Supplementary Fig. 5), Fe_3GaTe_2 bulk crystals show a clear out-of-plane magnetic anisotropy with in-plane saturation field B_{sat}=4 T and saturation magnetization M_{sat}=32.7 emu/g (0.3 T). The magnetic anisotropy energy density (K_u) is calculated as ~4.79×10^5 J/m^3 at 300 K, following the formula[1]:

$$K_u=\frac{B_{sat}M_{sat}}{2\mu_0} \quad (1)$$

where M_{sat} is the saturation magnetization, B_{sat} is the saturation field, and μ_0 is the permeability in free space.

The magnetic anisotropy of Fe_3GaTe_2 few-layer nanosheet (9.5 nm) is analyzed by angle-dependent anomalous Hall effect (AHE) at 300 K. The angle (θ_B) is defined as the tilt angle between the sample plane and the magnetic field (For example, θ_B=90° and θ_B=0° correspond to the perpendicular and parallel magnetic field, respectively). The square hysteresis loops suggest an out-of-plane magnetic anisotropy. An in-plane magnetic field (B>0.5 T, θ_B=0°) drives the magnetization to flip in an in-plane direction, R_{xy} disappears because it is proportional only to the out-of-plane component of the magnetization. To eliminate the effect of domains, the R_{xy} value is extracted at high magnetic field (B=4 T), and the angle between magnetization and the sample plane, θ_M, is calculated by using the formula[2]:

$$\theta_M(\theta_B)=arcsin\left[\frac{R_{xy}(\theta_B)}{R_{xy}(\theta_B=90°)}\right] \quad (2)$$

The magnetic anisotropy energy density K_u is estimated by fitting the data of θ_B and θ_M with the Stoner-Wohlfarth model[3]. The total energy of the system is:

$$E=K_u sin^2(\theta_M)-H_k M_{sat} cos(\theta_B-\theta_M) \quad (3)$$

At first-order derivative:

$$\frac{\partial E}{\partial \theta_M}=2K_u sin(\theta_M) cos(\theta_M)+H_k M_{sat} sin(\theta_B-\theta_M)=0 \quad (4)$$

The fitting curve in Fig. 2g (main text) is obtained from the above equation, and the K_u is calculated as ~3.88×10^5 J/m^3.

Supplementary Note 3. Additional magneto-transport analysis of the single-sheet Fe_3GaTe_2 nanosheets

The AHE measurements are performed under the perpendicular magnetic field at a series of temperature. In a ferromagnetic material with a uniaxial out-of-plane magnetic anisotropy, the R_{xy} can be expressed by the following formula:

$$R_{xy}=R_0\mu_0H+R_sM \quad (5)$$

where R_0 and R_S are normal and anomalous Hall coefficients, respectively, and M is the magnetization. For μ_0H=0, the remanent R_{xy}, namely R^r_{xy}, is only directly proportional to the zero-field magnetization, namely spontaneous magnetization. So the onset of non-zero R^r_{xy} reflect the emergence of spontaneous magnetization, and further demonstrates the long-range ferromagnetic order (or intrinsic ferromagnetism) in Fe_3GaTe_2 nanosheet. Therefore, the T_C is determined at the onset of non-zero R^r_{xy}.[2]

In Supplementary Fig. 10, the normal Hall coefficient (R_0), anomalous Hall coefficient (R_s), carrier density (n), and mobility (μ) are calculated by using the formulas:

$$R_0=\frac{\Delta R_{xy}}{\Delta B}*d \quad (6)$$

$$R_s=\frac{\rho_{xy}(0)}{M(0)} \quad (7)$$

$$n=\frac{1}{eR_0} \quad (8)$$

$$\mu=R_0\times\sigma_{xx} \quad (9)$$

where $M(0)$ is the zero-field magnetization, σ_{xx} is the longitudinal conductivity, $e\approx1.602\times10^{-19}$ C.

In Supplementary Fig. 11, the anomalous Hall conductivity (σ_{AH}) and anomalous Hall angle (θ_{AH}) are calculated by using the formulas:

$$\sigma_{AH}=\frac{\rho_{AH}}{\rho_{AH}^2+\rho_{xx}^2} \quad (10)$$

$$\theta_{AH}=\frac{\sigma_{AH}}{\sigma_{xx}}\times100\% \quad (11)$$

where ρ_{AH} is the anomalous Hall resistivity, ρ_{xx} is the longitudinal resistivity, σ_{xx} is the longitudinal conductivity.

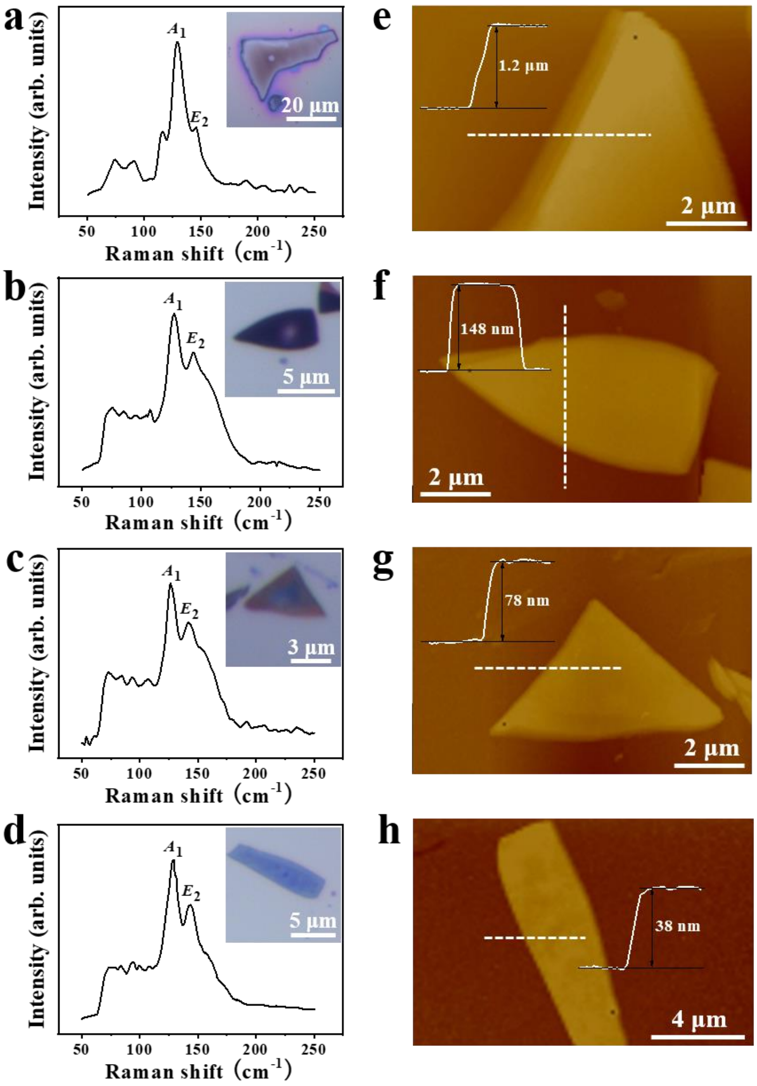

Supplementary Fig. 1. Raman characterization of the Fe_3GaTe_2 nanosheets with different thickness. (**a-d**) Raman spectra of the Fe_3GaTe_2 nanosheets with different thickness. Insets show the corresponding optical micrographs. (**e-h**) Corresponding AFM topographies and thickness of the as-tested Fe_3GaTe_2 nanosheets.

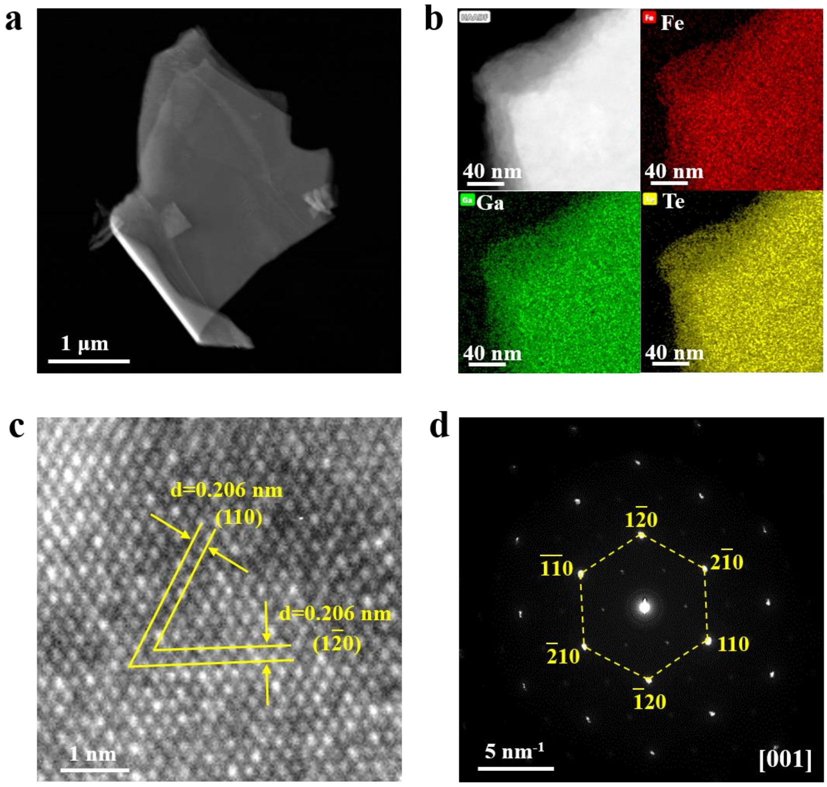

Supplementary Fig. 2. TEM characterization of a Fe_3GaTe_2 nanosheet. (**a, b**) Dark-field image and corresponded high-resolution element mapping image of a Fe_3GaTe_2 nanosheet. (**c, d**) HRTEM image and corresponded SAED pattern of the Fe_3GaTe_2 nanosheet.

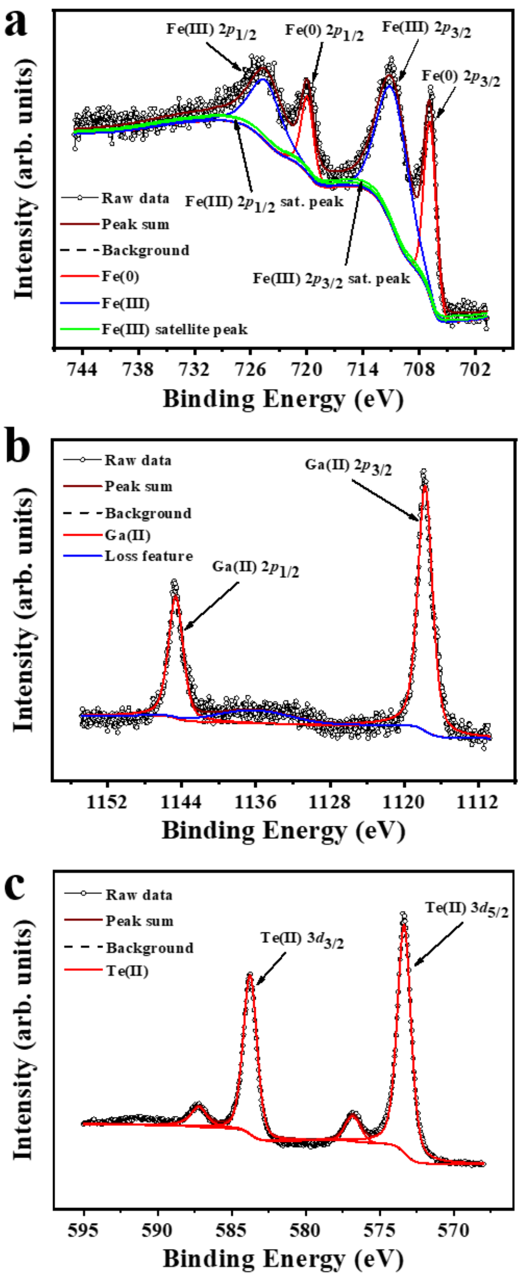

Supplementary Fig. 3. XPS analysis of the exfoliated fresh Fe_3GaTe_2 crystal surface. (**a**) Fe. (**b**) Ga. (**c**) Te.

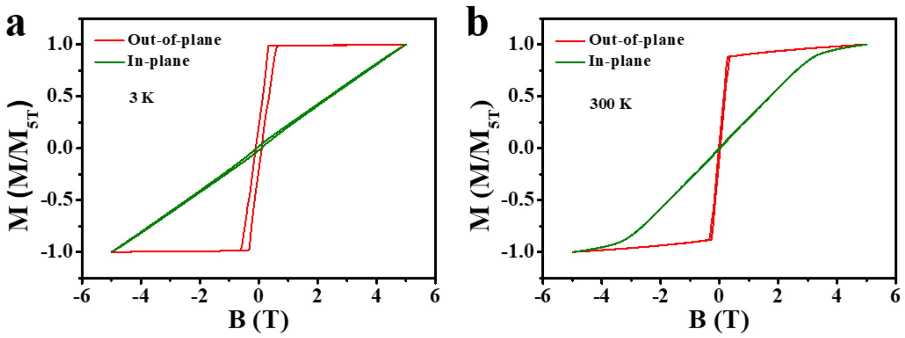

Supplementary Fig. 4. Magnetic anisotropy of Fe_3GaTe_2 bulk crystals by VSM. (**a**, **b**) M-H curves for out-of-plane and in-plane orientation with normalized moment at 3 K (**a**) and 300 K (**b**).

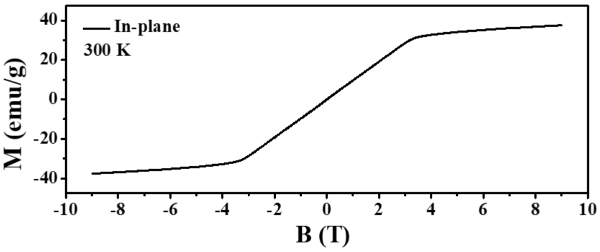

Supplementary Fig. 5. M-H curve of the Fe_3GaTe_2 bulk crystals under in-plane magnetic field of -9 to 9T at 300 K.

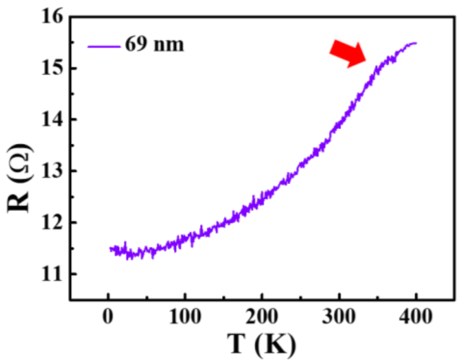

Supplementary Fig. 6. Temperature-dependent longitudinal resistance (R-T) curve of a 69 nm Fe_3GaTe_2 nanosheet from 3 to 400 K. The red arrow shows a kink, which are characteristic of the established phase transition from paramagnetism to ferromagnetism.

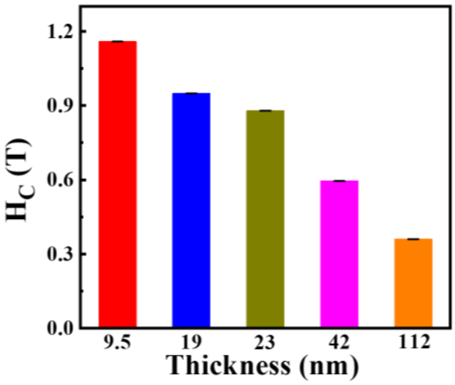

Supplementary Fig. 7. Thickness-dependent H_C histogram of Fe_3GaTe_2 nanosheets at 3 K. The data are obtained from Fig. 3d in main text. Error bars s.d., N=25.

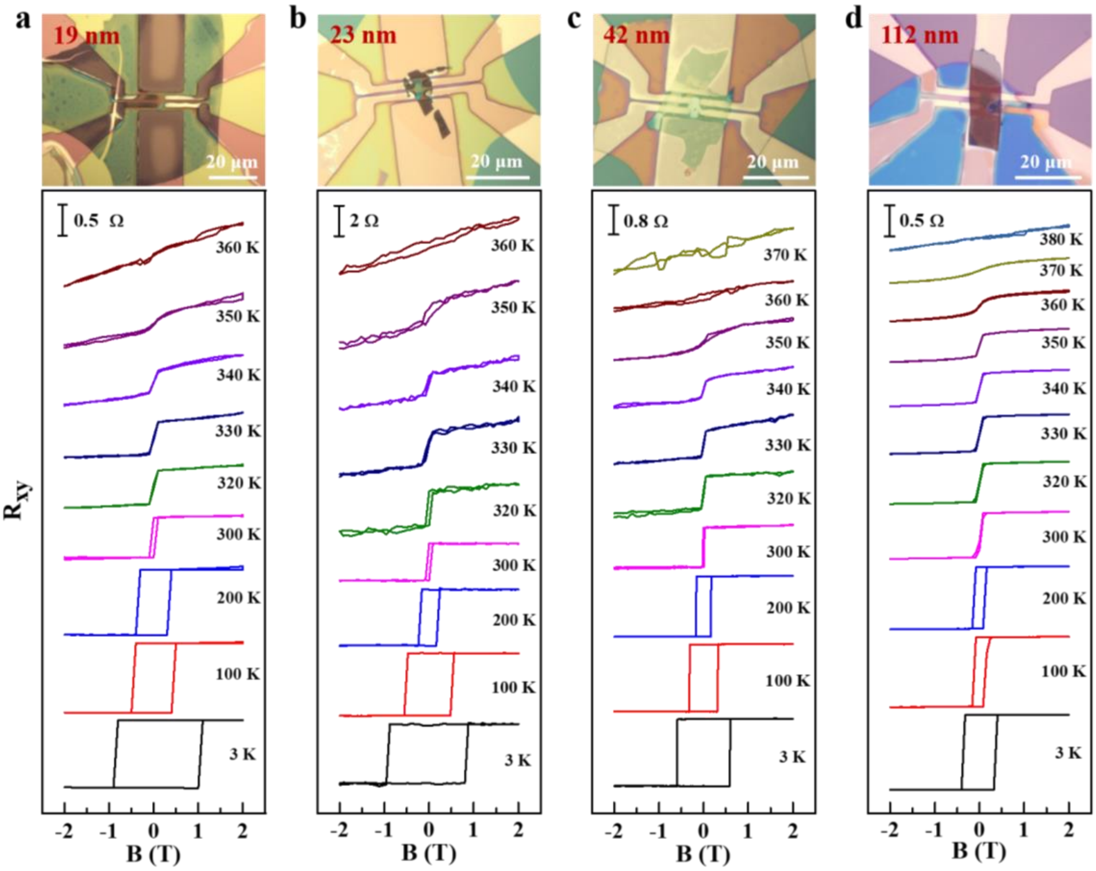

Supplementary Fig. 8. Optical images of 19 (a), 23 (b), 42 (c) and 112 nm (d) Fe_3GaTe_2 Hall devices and corresponded temperature-dependent AHE.

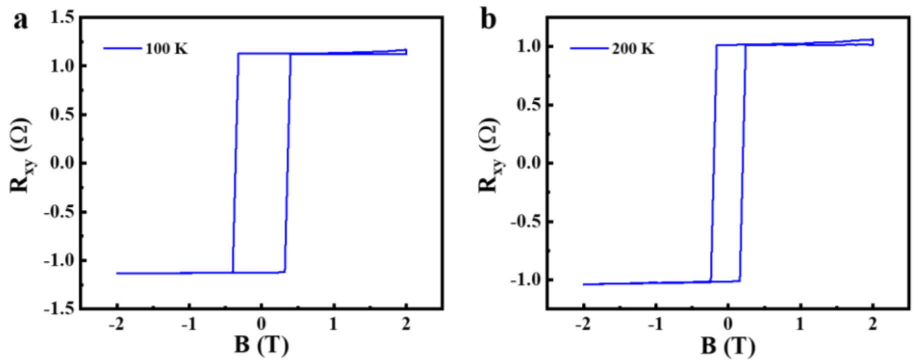

Supplementary Fig. 9. Additional AHE data in 9.5 nm few-layer Fe_3GaTe_2 nanosheet at 100 K (a) and 200 K (b). Other AHE data at 3, 300, 320-350 K can be found in Fig. 3d, 3e, 3f in main text, respectively.

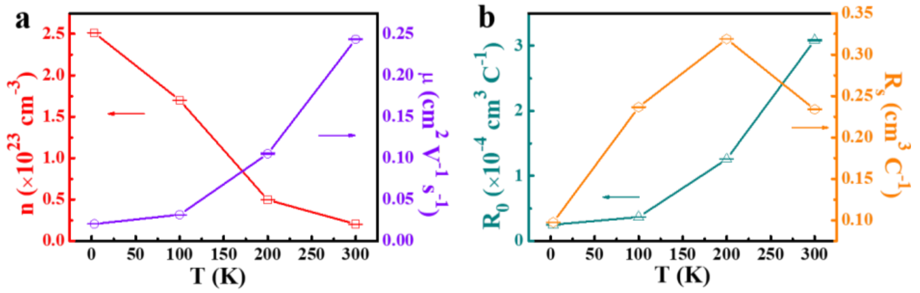

Supplementary Fig. 10. Basic parameter of a 9.5 nm Fe_3GaTe_2 few-layer nanosheet from magneto-transport measurement. (**a**) Temperature-dependent carrier density (*n*) and mobility (*μ*). (**b**) Temperature-dependent normal Hall coefficient (R_0) and anomalous Hall coefficient (R_s). Error bars s.d., N=25. See data in Supplementary Table 5.

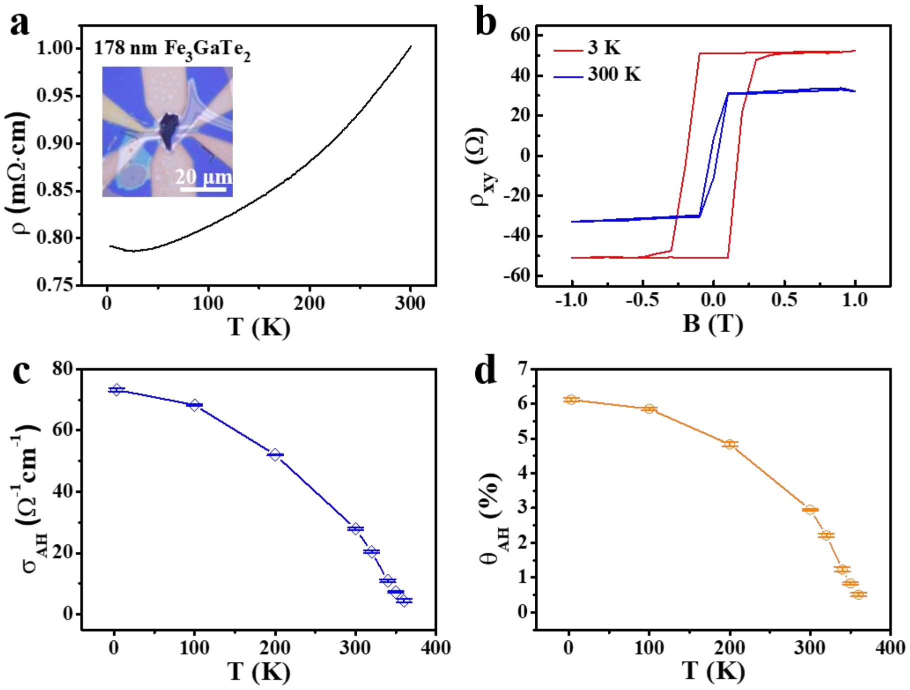

Supplementary Fig. 11. Magneto-transport measurement of a 178 nm Fe_3GaTe_2 nanosheet. (**a**) Temperature-dependent longitudinal resistivity (ρ-T) curve. Inset shows the optical image of the as-tested Fe_3GaTe_2 Hall device. (**b**) Temperature-dependent magnetic field (out-of-plane) sweeps of the Hall resistivity (ρ_{xx}) at 3 K and 300 K. (**c, d**) Temperature-dependent anomalous Hall conductivity (σ_{AH}) (**c**) and anomalous Hall angle (θ_{AH}) (**d**). Error bars s.d., N=25.

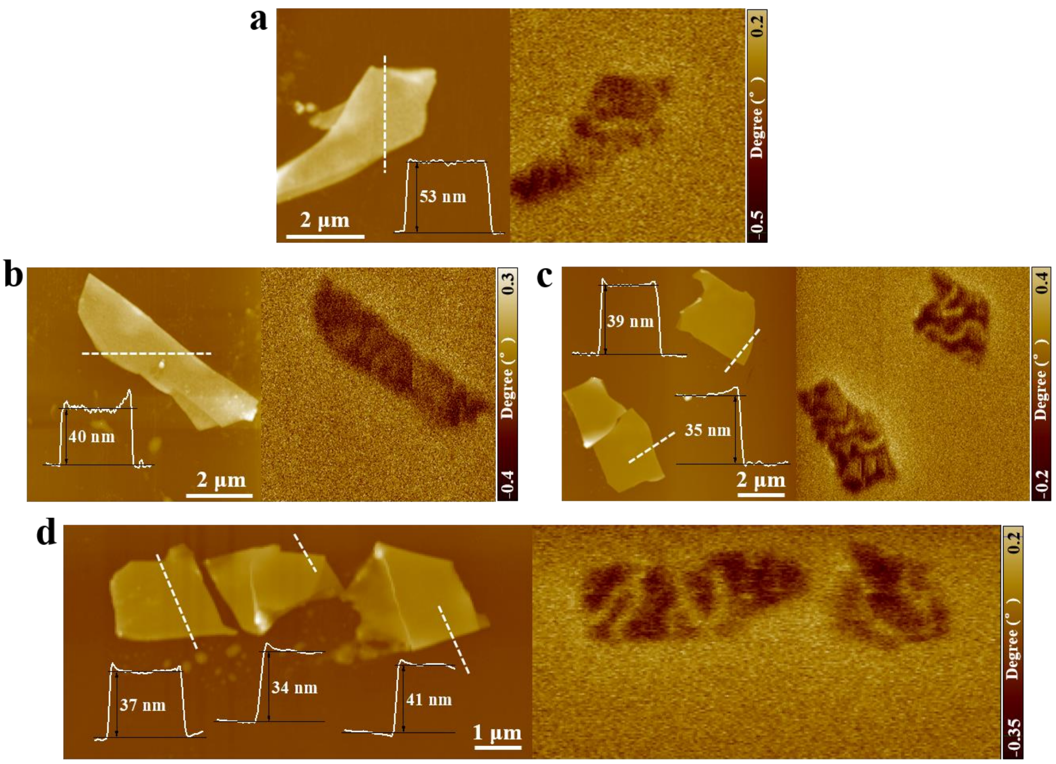

Supplementary Fig. 12. Direct imaging intrinsic ferromagnetism and magnetic domains of Fe_3GaTe_2 nanosheets with different thickness at 300 K by MFM without external magnetic field.

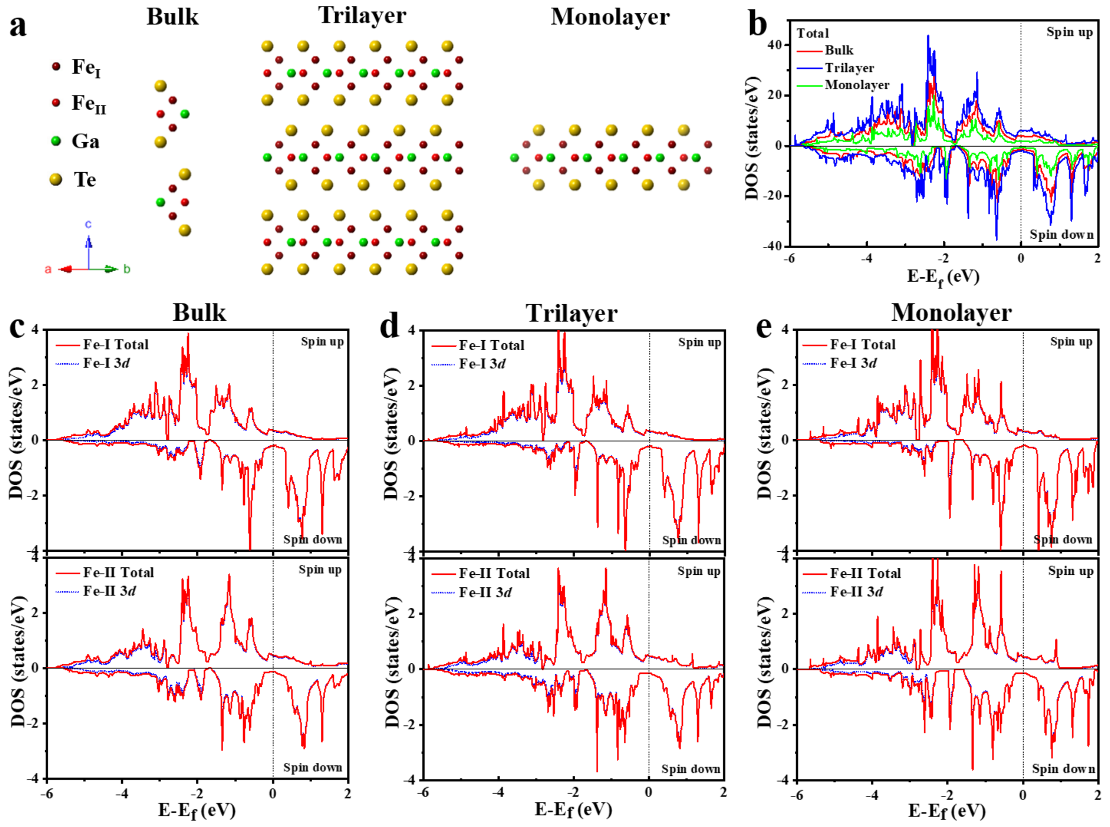

Supplementary Fig. 13. Spin-resolved density of states (DOSs) of the vdW Fe_3GaTe_2 ferromagnetic bulk and few-layer crystals. (**a**) Supercells of the bulk, trilayer and monolayer vdW Fe_3GaTe_2 crystals. (**b**) Total DOSs in bulk, trilayer and monolayer vdW Fe_3GaTe_2 crystals. (**c-e**) Total and partial DOSs of two types of Fe 3*d* states in bulk (**c**), trilayer (**d**) and monolayer (**e**) vdW Fe_3GaTe_2 crystals. The vertical dash lines denote the position of Fermi level.

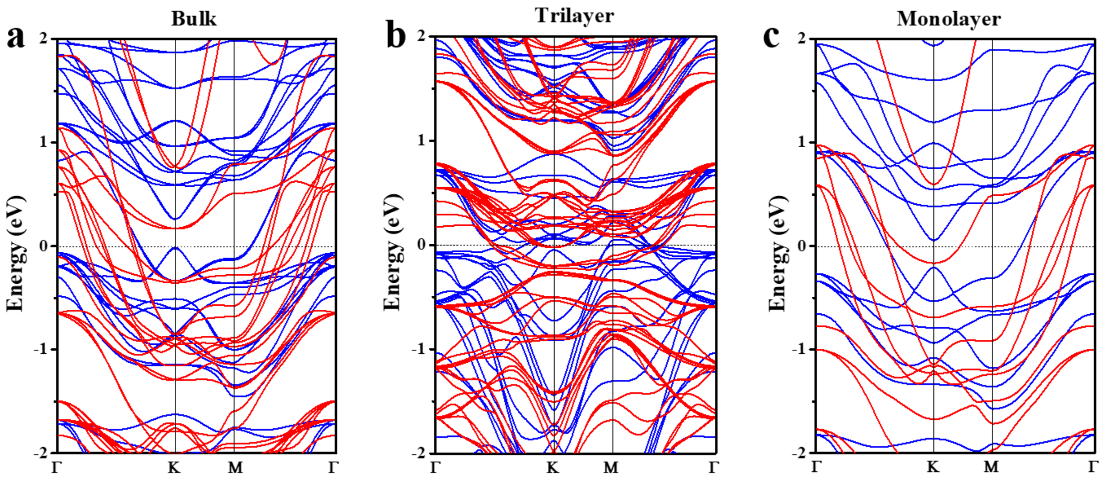

Supplementary Fig. 14. Spin-resolved band structures of the bulk (a), trilayer (b) and monolayer (c) vdW Fe_3GaTe_2 ferromagnetic bulk and few-layer crystals. The red and blue solid lines represent the majority-spin and minority-spin bands, respectively. The horizontal dash lines denote the position of Fermi level.

Supplementary Table 1. Element analysis of the Fe_3GaTe_2 by EDS and XPS measurements.

Method	Elements	Atomic Conc (%)	Error (%)
EDS-1	Fe K	48.75	-
	Ga K	18.95	-
	Te K	32.30	-
EDS-2	Fe K	51.01	-
	Ga K	16.52	-
	Te K	32.47	-
EDS-3	Fe K	50.09	-
	Ga K	17.09	-
	Te K	32.82	-
EDS-Ave	Fe K	49.95	Sd. 0.93
	Ga K	17.52	Sd. 1.04
	Te K	32.53	Sd. 0.22
XPS	Fe 2*p*	49.90	3.02
	Ga 2*p*	17.60	1.44
	Te 3*d*	32.50	2.02

Notes for Supplementary Table 1:

For the EDS measurement, we measured 3 points on an exfoliated Fe_3GaTe_2 thin nanosheet. The average atomic ratio of Fe: Ga: Te was 2.94±0.05: 1.03±0.06: 1.91±0.01, which is very close to 3: 1: 2. Additionally, we also performed the XPS measurement on an exfoliated fresh Fe_3GaTe_2 crystal surface. The atomic ratio of Fe: Ga: Te was 2.94: 1.04: 1.91, which is also very close to 3: 1: 2. The above EDS and XPS measurements were performed in the vacuum TEM and XPS chambers, respectively.

Supplementary Table 2. Saturation magnetic moment (*M_{sat}*) and coercivity (*H_C*) of bulk Fe_3GaTe_2 with out-of-plane magnetic field at different temperatures.

T (K)	*M_{sat}* (emu/g)	*M_{sat}* (μB/Fe)	*H_C* (Oe)
3	57.18 (± 3.12×10^{-4})	1.68 (± 2.75×10^{-5})	1014.19 (± 0.62)
100	55.39 (± 5.29×10^{-4})	1.63 (± 4.67×10^{-5})	378.23 (± 0.09)
200	50.15 (± 1.09×10^{-4})	1.47 (± 9.61×10^{-5})	287.87 (± 0.36)
300	40.11 (± 2.82×10^{-4})	1.18 (± 2.49×10^{-5})	248.71 (± 0.58)
350	31.05 (± 3.33×10^{-4})	0.91 (± 2.94×10^{-5})	209.13 (± 0.17)

Supplementary Table 3. Ferromagnetic properties of Fe_3GaTe_2 in comparison with other vdW intrinsic ferromagnetic crystals.

Materials	Type	Easy axis	M_{sat} (uB)	T_C (K)	Ref.
CrI_3	Semi	OOP	3.1 @ 2K&6T	61	[4]
$Cr_2Ge_2Te_6$	Semi	OOP	2.39 @ 2K&5T	66	[5]
			2.4 @ 5K&1T	61	[6]
$CrSiTe_3$	Semi	OOP	3.08 @ 2K&6T	33	[7]
$CrBr_3$	Semi	OOP	2.85 @ 2K&2T	33	[8]
VI_3	Semi	OOP	1.25 @ 1.8K&9T	49	[9]
			2.47 @ 1.8K&5T	50	[10]
			1.31 @ 2K&7T	50	[11]
$AgVP_2Se_6$	Semi	OOP	1.38 @ 2K&1.5T	18.5	[12]
$CrTe_2$	Metal	IP	1.81 @ 2K&7T	310	[13]
$Cr_{1.33}Te_2$	Metal	OOP	2.66 @ 3K&7T	312	[14]
Fe_3GeTe_2	Metal	OOP	1.6 @ 10K&5T	220	[15]
Fe_4GeTe_2	Metal	OOP	1.8 @ 2K&1T	270	[16]
Fe_5GeTe_2	Metal	IP	2.1 @ 3K&6T	317	[17]
$Fe_{1/4}TaS_2$	Metal	OOP	3 @ 2K&7T	160	[18]
$Cr_{1/3}TaS_2$	Metal	IP	1.85 @ 10K&5T	110	[19]
$Cr_{1/3}NbS_2$	Metal	IP	2.9 @ 4.2K&1.5T	127	[20]
$Mn_{1/3}TaS_2$	Metal	IP	2.68 @ 2K&6T	70	[21]
$Ni_2FeGaTe_2$	Metal	-	0.6 @ 4K&5T	75	[22]
$Fe_{2.7}AsTe_2$	Metal	-	0.1 @ 2K&7T	123	[23]
$Fe_{2.9}Ge_{1-x}As_xTe_2$	Metal	-	1.1-0.25 @ 10K&5T	177-33	[24]
(Fe,Ni)$GeTe_2$	Metal	-	2.11 @ 1.8K&5T	279	[25]
**Fe_3GaTe_2**	**Metal**	**OOP**	**1.68 @ 3K&5T**	**367**	**This work**

Notes for Supplementary Table 3:

OOP is out-of-plane, IP is in-plane. Fe_3GaTe_2 possess record-high T_C compared with other vdW intrinsic ferromagnets, which is significant for room-temperature vdW-integrated spintronic device. This table only compares data for bulk crystals.

Supplementary Table 4. Comparison of magnetic anisotropy energy density K_u in some classical PMA systems at room temperature from literatures.

Structure	Materials	Sample Information	K_u ($\times 10^5$ J/m^3)	Ref.
Non-vdW	CoFeB	1.3 nm thin film	2.10	[1]
	Co_2FeAl	2 nm thin film	1.30	[26]
	Fe_2CrSi	0.8 nm thin film	2.80	[27]
VdW	$CrTe_2$	7 layers thin film	0.49	[28]
	Cr-$CrTe_2$	Bulk crystals	0.76	[14]
	**Fe_3GaTe_2**	**Bulk crystals**	**4.79**	**This**
		**9.5 nm nanosheet**	**3.88**	**work**

Notes for Supplementary Table 4:

The K_u in Fe_3GaTe_2 is comparable to the conventional ferromagnetic thin film and one order of magnitude larger than recently discovered vdW ferromagnetic crystals. The large K_u value (~4.79×10^5 J/m^3 for bulk crystals and ~3.88×10^5 J/m^3 for 2D few-layer nanosheet) in Fe_3GaTe_2 indicate the strong and robust PMA, which is of crucial importance to maintain high thermal stability for long-term data retention in high-density magnetic data-storage devices.

Supplementary Table 5. Normal Hall coefficient (R_0), anomalous Hall coefficient (R_s), conductivity (σ), carrier density (n) and mobility (μ) of the 9.5 nm Fe_3GaTe_2 few-layer nanosheet at different temperatures.

T (K)	R_0 (cm^3 C^{-1})	R_s (cm^3 C^{-1})	σ (S cm^{-1})	n (cm^{-3})	μ (cm^2 V^{-1} s^{-1})
3	2.49×10^{-5}	0.09759	830.74	2.51×10^{23}	0.02
100	3.68×10^{-5}	0.23664	861.07	1.70×10^{23}	0.03
200	1.26×10^{-4}	0.3189	838.35	4.98×10^{22}	0.11
300	3.09×10^{-4}	0.23396	789.30	2.03×10^{22}	0.24

Supplementary Table 6. Electric conductivity (σ_{xx}) and anomalous Hall angle (θ_{AH}) of Fe_3GaTe_2 in comparison with other ferromagnets.

Materials	Sample Information	σ_{xx} (10^4 Ω^{-1} cm^{-1})	θ_{AH} (%)	Ref.
$Co_3Sn_2S_2$	Single crystal, 130 K	0.57	19.8	29
Fe_3GeTe_2	Single crystal, 5 K	0.67	9.0	30
$Fe_{0.28}TaS_2$	Single crystal, 4.2 K	0.90	3.7	31
(Ga, Mn)As	50 nm thin film, 2 K	0.22	6.6	32
Fe-Co alloy	0.25 wt% Co-Fe, 4.2 K	438.00	1.5	33
$Fe_{0.99}Co_{0.01}$	Bulk, 5 K	110.00	1.2	34
Fe_3Sn_2	Single crystal, 10 K	12.50	1.0	35
Fe	Thin film, 5 K	4.36	2.6	36
Gd	Thin film, 5 K	50.00	0.2	36
$CrTe_2$	170 nm, 2 K	133.08	5.5	37
	170 nm, 300 K	<1.00	<0.5	
**Fe_3GaTe_2**	**178 nm, 3 K**	**0.12**	**6.1**	**This work**
	**178 nm, 300 K**	**0.09**	**3.0**	

Supplementary Table 7. Calculated atomic magnetic moment (μB) of the bulk, trilayer and monolayer Fe_3GaTe_2.

Element	Bulk	Trilayer	Monolayer
Fe-I	2.356	2.327	2.373
Fe-II	1.459	1.454	1.421
Ga	-0.114	-0.112	-0.113
Te	-0.09	-0.088	-0.089

Supplementary References

1. Ikeda S, *et al.* A perpendicular-anisotropy CoFeB-MgO magnetic tunnel junction. *Nat. Mater.* **9**, 721-724 (2010).
2. Yujun Deng, *et al.* Gate-tunable room-temperature ferromagnetism in two-dimensional Fe_3GeTe_2. *Nature* **563**, 94-99 (2018).
3. Stoner EC, Wohlfarth EP. A mechanism of magnetic hysteresis in heterogeneous alloys. *Phil. Trans. R. Soc. Lond. A* **240**, 599-642 (1948).
4. McGuire MA, Dixit H, Cooper VR, Sales BC. Coupling of Crystal Structure and Magnetism in the Layered, Ferromagnetic Insulator CrI_3. *Chem. Mater.* **27**, 612-620 (2015).
5. Liu Y, Petrovic C. Critical behavior of quasi-two-dimensional semiconducting ferromagnet $Cr_2Ge_2Te_6$. *Phys. Rev. B* **96**, 054406 (2017).
6. Xing W, *et al.* Electric field effect in multilayer $Cr_2Ge_2Te_6$: a ferromagnetic 2D material. *2D Mater.* **4**, 024009 (2017).
7. Casto LD, *et al.* Strong spin-lattice coupling in $CrSiTe_3$. *APL Mater.* **3**, 041515 (2015).
8. Yu X, *et al.* Large magnetocaloric effect in van der Waals crystal $CrBr_3$. *Frontiers of Physics* **14**, 43501 (2019).
9. Kong T, *et al.* VI_3-a New Layered Ferromagnetic Semiconductor. *Adv. Mater.* **31**, e1808074 (2019).
10. Tian S, *et al.* Ferromagnetic van der Waals Crystal VI_3. *J. Am. Chem. Soc.* **141**, 5326-5333 (2019).
11. Son S, *et al.* Bulk properties of the van der Waals hard ferromagnet VI_3. *Phys. Rev. B* **99**, 041402 (2019).
12. Peng Y, *et al.* A Quaternary van der Waals Ferromagnetic Semiconductor $AgVP2Se_6$. *Adv. Funct. Mater.* **30**, 1910036 (2020).
13. Sun X, *et al.* Room temperature ferromagnetism in ultra-thin van der Waals crystals of 1T-$CrTe_2$. *Nano Res.* **13**, 3358-3363 (2020).
14. Huang M, *et al.* Significant perpendicular magnetic anisotropy in room-temperature layered ferromagnet of Cr-intercalated $CrTe_2$. *2D Mater.* **8**, 031003 (2021).
15. Chen B, *et al.* Magnetic Properties of Layered Itinerant Electron Ferromagnet Fe_3GeTe_2. *J. Phys. Soc. Japan* **82**, 124711 (2013).
16. Junho Seo, *et al.* Nearly room temperature ferromagnetism in a magnetic metal-rich van der Waals metal. *Sci. Adv.* **6**, eaay8912 (2020).
17. Zhang H, *et al.* Itinerant ferromagnetism in van der Waals $Fe_{5-x}GeTe_2$ crystals above room temperature. *Phys. Rev. B* **102**, 064417 (2020).
18. Chen C-W, Chikara S, Zapf VS, Morosan E. Correlations of crystallographic defects and anisotropy with magnetotransport properties in Fe_xTaS_2 single crystals ($0.23 \leq x \leq 0.35$). *Phys. Rev. B* **94**, 054406 (2016).
19. Yamasaki Y, *et al.* Exfoliation and van der Waals heterostructure assembly of intercalated ferromagnet $Cr_{1/3}TaS_2$. *2D Mater.* **4**, 041007 (2017).
20. Tomonao Miyadai, Katsuya Kikuchi, Hiromitsu Kondo, Shuzo Sakka, Masatoshi Arai, Ishikawa Y. Magnetic properties of $Cr_{1/3}NbS_2$. *J. Phys. Soc. Jpn.* **52**, 1394

(1983).
21. Zhang H, *et al.* Electrical and anisotropic magnetic properties in layered $Mn_{1/3}TaS_2$ crystals. *Appl. Phys. Lett.* **113**, 072402 (2018).
22. Kuznetsov AN, *et al.* Mixed nickel-gallium tellurides $Ni_{3-x}GaTe_2$ as a matrix for incorporating magnetic cations: A $Ni_{3-x}FexGaTe_2$ series. *J. Solid. State. Chem.* **250**, 90-99 (2017).
23. Verchenko VY, *et al.* New Fe-based layered telluride $Fe_{3-\delta}As_{1-y}Te_2$: synthesis, crystal structure and physical properties. *Dalton Trans* **45**, 16938-16947 (2016).
24. Yuan D, *et al.* Tuning magnetic properties in quasi-two-dimensional ferromagnetic $Fe_{3-y}Ge_{1-x}As_xTe_2$ ($0 \leq x \leq 0.85$). *Mater. Res. Express.* **4**, 036103 (2017).
25. Stahl J, Shlaen E, Johrendt D. The van der Waals Ferromagnets $Fe_{5-\delta}GeTe_2$ and $Fe_{5-\delta-x}Ni_xGeTe_2$-Crystal Structure, Stacking Faults, and Magnetic Properties. *Z. Anorg. Allg. Chem.* **644**, 1923-1929 (2018).
26. Xiaoqi Li, *et al.* Perpendicular Magnetic Anisotropy of Full-Heusler Films in Pt/Co_2FeAl/MgO Trilayer. *Appl. Phys. Express* **4**, 043006 (2011).
27. Wang Y-P, Qiu J-J, Lu H, Ji R, Han G-C, Teo K-L. Perpendicular magnetic anisotropy in $Fe_2Cr_{1-x}Co_xSi$ Heusler alloy. *J. Phys. D* **47**, 495002 (2014).
28. Zhang X, *et al.* Room-temperature intrinsic ferromagnetism in epitaxial $CrTe_2$ ultrathin films. *Nat. Commun.* **12**, 2492 (2021).
29. Liu E, *et al.* Giant anomalous Hall effect in a ferromagnetic Kagome-lattice semimetal. *Nat. Phys.* **14**, 1125-1131 (2018).
30. Wang Y, *et al.* Anisotropic anomalous Hall effect in triangular itinerant ferromagnet Fe_3GeTe_2. *Phys. Rev. B* **96**, 134428 (2017).
31. Dijkstra J, Zijlema P, Van Bruggen C, Haas C, De Groot R. Band-structure calculations of $Fe_{1/3}TaS_2$ and $Mn_{1/3}TaS_2$, and transport and magnetic properties of $Fe_{0.28}TaS_2$. *J. Phys.: Condens. Matter* **1**, 6363 (1989).
32. Pu Y, Chiba D, Matsukura F, Ohno H, Shi J. Mott relation for anomalous Hall and Nernst effects in $Ga_{1-x}Mn_xAs$ ferromagnetic semiconductors. *Phys. Rev. Lett.* **101**, 117208 (2008).
33. Majumdar AK, Berger L. Hall Effect and Magnetoresistance in Pure Iron, Lead, Fe-Co, and Fe-Cr Dilute Alloys. *Phys. Rev. B* **7**, 4203-4220 (1973).
34. Shiomi Y, Onose Y, Tokura Y. Extrinsic anomalous Hall effect in charge and heat transport in pure iron,$Fe_{0.997}Si_{0.003}$, and $Fe_{0.97}Co_{0.03}$. *Phys. Rev. B* **79**, 100404 (2009).
35. Ye L, *et al.* Massive Dirac fermions in a ferromagnetic kagome metal. *Nature* **555**, 638-642 (2018).
36. Miyasato T, *et al.* Crossover behavior of the anomalous Hall effect and anomalous nernst effect in itinerant ferromagnets. *Phys. Rev. Lett* **99**, 086602 (2007).
37. Huang M, *et al.* Colossal Anomalous Hall Effect in Ferromagnetic van der Waals $CrTe_2$. *ACS Nano* **15**, 9759-9763 (2021).
